# Darunavir/Cobicistat/Emtricitabine/Tenofovir Alafenamide in a Rapid-Initiation Model of Care for Human Immunodeficiency Virus Type 1 Infection: Primary Analysis of the DIAMOND Study

**DOI:** 10.1093/cid/ciz1213

**Published:** 2019-12-27

**Authors:** Gregory D Huhn, Gordon Crofoot, Moti Ramgopal, Joseph Gathe, Robert Bolan, Donghan Luo, Richard Bruce Simonson, Richard E Nettles, Carmela Benson, Keith Dunn

**Affiliations:** 1 Ruth M. Rothstein CORE Center, Chicago, Illinois, USA; 2 Crofoot Research Center, Houston, Texas, USA; 3 Midway Immunology and Research Center, Fort Pierce, Florida, USA; 4 Therapeutic Concepts, Houston, Texas, USA; 5 Los Angeles LGBT Center, Los Angeles, California, USA; 6 Janssen Research & Development, LLC, Titusville, New Jersey, USA; 7 Janssen Scientific Affairs, LLC, Titusville, New Jersey, USA

**Keywords:** rapid initiation, darunavir, D/C/F/TAF, single-tablet regimen, HIV-1

## Abstract

**Background:**

Most guidelines recommend rapid treatment initiation for patients with newly diagnosed human immunodeficiency virus type 1 (HIV-1) infection, but prospective US data are limited. The DIAMOND (NCT03227861) study using darunavir/cobicistat/emtricitabine/tenofovir alafenamide (D/C/F/TAF) 800/150/200/10 mg is a phase 3 prospective study evaluating efficacy/safety of a single-tablet regimen in a rapid-initiation model of care.

**Methods:**

Adults aged ≥18 years began D/C/F/TAF ≤14 days from diagnosis without screening/baseline results; as results became available, participants not meeting predefined safety/resistance stopping rules continued. Primary endpoint was virologic response (HIV-1 RNA <50 copies/mL; intent-to-treat; US Food and Drug Administration [FDA] snapshot) at week 48; participant satisfaction was measured via the HIV Treatment Satisfaction Questionnaire status version (HIVTSQs).

**Results:**

Of 109 participants, 87% were male, 32% black/African American, median (range) age was 28 (range, 19–66) years, 25% of participants had HIV-1 RNA ≥100 000 copies/mL, 21% had CD4^+^ cell count <200 cells/µL, and 31% enrolled ≤48 hours from diagnosis. At week 48, 97 (89%) participants completed the study and 92 (84%) achieved HIV-1 RNA <50 copies/mL (FDA snapshot). There were no protocol-defined virologic failures; incidences of adverse events (AEs) and adverse drug reactions (33%) were low, no serious AEs were study drug related, and 1 (<1%) participant discontinued due to study drug related AE(s). The overall HIVTSQs score at week 48 was 58 (maximum: 60).

**Conclusions:**

At week 48, a high proportion of participants starting D/C/F/TAF achieved HIV-1 RNA <50 copies/mL and very few discontinued therapy. D/C/F/TAF was well tolerated, no participants discontinued due to baseline resistance stopping criteria, and high treatment satisfaction among participants was recorded.

**Clinical Trials Registration:**

NCT03227861.

## Teaser

Newly diagnosed patients with human immunodeficiency virus-1 (HIV-1) infection rapidly initiating treatment with darunavir/cobicistat/emtricitabine/tenofovir alafenamide demonstrated high rates of virologic suppression, few grade 3–4 and serious adverse events, no postbaseline HIV-1 resistance-associated mutations, and high treatment satisfaction after 48 weeks.

In 2016, only 64% of people living with human immunodeficiency virus type 1 (HIV-1) in the United States received care, 49% were retained in care, and 53% were virologically suppressed [1]. The US Department of Health and Human Services (DHHS) guidelines recommend that certain laboratory testing be performed to help guide initial treatment selection; some (eg, genotypic resistance testing, testing for HLA-B*5701) may require several days or weeks for results, which may contribute to patient attrition and delayed treatment [2]. In rapid-initiation models of care, therapy is started prior to the availability of baseline laboratory assessments, sometimes on the day of diagnosis [3, 4]. Improved retention, reduced time to virologic suppression, and decreased morbidity and mortality have been observed with this model in low-income countries and select US centers [5–10]. World Health Organization (WHO) and International Antiviral Society–USA guidelines recommend rapid initiation for the majority of newly diagnosed patients [3, 4]. Although the US DHHS considers this approach investigational, the guidelines recognize the importance of prompt antiretroviral therapy (ART) initiation for some patients [2]. Moreover, while US DHHS guidelines recommend an integrase inhibitor (INI)–based regimen as initial ART, in certain clinical situations such as those encountered when rapidly initiating therapy, a boosted protease inhibitor (PI)–based regimen is a recommended option [2].

As less clinical information is available in a rapid-initiation model of care, it is important to consider a regimen’s effectiveness in the setting of possible transmitted resistance, safety profile, and convenience. The potential for adherence is particularly meaningful, as patients newly diagnosed with HIV-1 infection may be hesitant to rapidly begin ART due to concerns regarding the need for lifelong therapy, side effects, and dosing requirements, as well as psychological considerations associated with the diagnosis [3]. An optimal ART regimen for rapid initiation is an abacavir-sparing, single-tablet regimen (STR) that is well tolerated and has a proven high genetic barrier to resistance. No prospective clinical study of rapid initiation with such a regimen has been conducted to date. Furthermore, data on patient-reported outcomes (PROs) are lacking in rapid-initiation scenarios.

Darunavir/cobicistat/emtricitabine/tenofovir alafenamide (D/C/F/TAF) 800/150/200/10 mg is an oral, once-daily STR for treatment of naïve and ART-experienced, virologically suppressed patients with HIV-1 infection. The efficacy and safety of D/C/F/TAF have been demonstrated in the phase 3 AMBER and EMERALD studies, in which high proportions of participants (>91%) achieved HIV-1 RNA <50 copies/mL [11, 12]. Few participants (<2% in each study) had adverse events (AEs) leading to study discontinuation, and only 1 participant (of 1866 in total) had an emergent resistance-associated mutation (RAM) to a study drug (emtricitabine [AMBER]). Darunavir has demonstrated a high genetic barrier to resistance and is recommended for cases in which resistance testing records are unavailable, when ART needs to be started prior to the availability of resistance testing results, or when poor adherence is suspected [2, 13]. In the DIAMOND study, D/C/F/TAF was prospectively assessed in a rapid-initiation scenario in newly diagnosed, HIV-1–infected, treatment-naïve participants.

## METHODS

### Study Design and Population

DIAMOND (ClinicalTrials.gov identifier: NCT03227861) was a phase 3, open-label, single-arm, prospective, multicenter, 48-week study evaluating D/C/F/TAF rapid initiation (the study design is summarized in Supplementary Figure 1). The 16 study sites were strategically selected to target a diverse population [14]. Key inclusion criteria were as follows: adults aged ≥18 years; newly diagnosed with HIV-1 infection ≤2 weeks from the screening/baseline visit; and ART naïve. Key exclusion criteria were presence of opportunistic infections or AIDS-defining conditions that would preclude immediate ART initiation, and certain clinically relevant renal and hepatic conditions. Additional eligibility criteria are provided in the Supplementary Materials (page 1).

Participants who met eligibility requirements were enrolled and started on D/C/F/TAF within 24 hours of the screening/baseline visit, prior to the availability of laboratory results. Screening/baseline laboratory findings were reviewed as they became available. Participants not meeting predefined safety or resistance stopping rules continued treatment; those who met the stopping criteria discontinued and transitioned to care outside of the study protocol. Screening/baseline safety laboratory results were evaluated on day 3; safety stopping criteria are described in the Supplementary Materials (pages 1–2). Antiretroviral resistance results collected at baseline were evaluated at week 4 based on predicted genotypic sensitivity (assessed using GenoSure PRIme assay; there was no exclusion based on the presence of specific RAMs). Participants not showing full sensitivity to all D/C/F/TAF components were required to stop, with the exception of participants with resistance to lamivudine/emtricitabine associated with the M184I or M184V mutation alone. Every reasonable effort was made to contact participants missing study visits prior to counting them as lost to follow-up.

The trial was conducted in accordance with the principles of Good Clinical Practice and the Declaration of Helsinki. The protocol was approved by the Sterling Institutional Review Board (IRB) and all contributing sites that required local IRB approval. All participants provided written informed consent.

### Analyses

The primary endpoint was the proportion of participants with virologic response at week 48 (visit window: weeks 42–54), defined as HIV-1 RNA <50 copies/mL (US Food and Drug Administration [FDA] snapshot). The proportion of participants with HIV-1 RNA <50 copies/mL or <200 copies/mL using the observed algorithm (excluding participants with missing data) was also assessed.

Postbaseline samples were eligible for resistance testing using the Phenosense GT assay in participants with HIV-1 RNA values ≥400 copies/mL and protocol-defined virologic failure (PDVF; defined in the Supplementary Materials [page 2]).

Safety was assessed by discontinuations due to protocol-defined safety stopping rules, AEs, adverse drug reactions (ADRs; defined as AEs at least possibly related to the study drug), and laboratory abnormalities. PROs for treatment satisfaction were evaluated at weeks 4, 24, and 48 using the validated, 10-item HIV Treatment Satisfaction Questionnaire status version (HIVTSQs) [15].

For additional methodological details, including infection duration definitions, retention in care analyses, and HIVTSQs analyses, see the Supplementary Materials (pages 2–3).

### Statistical Analyses

Analyses were performed on all participants who received ≥1 dose of study drug (intent-to-treat [ITT] population). Descriptive statistics were used to calculate virologic response; missing values were not imputed.

## RESULTS

### Study Population

Of 109 participants enrolled in the study, all were included in the ITT population. The median age was 28 (range, 19–66) years, 87% of participants were men, 32% were black/African American, the median baseline body weight was 78.8 (range, 46–155) kg, and 75% had an HIV acquisition factor of men who have sex with men (MSM) (Table 1; see Supplementary Table 1 for participant geographic distribution). Overall, 25% of participants had HIV-1 RNA ≥100 000 copies/mL and 21% had a CD4^+^ cell count <200 cells/µL. The median time between HIV-1 diagnosis and screening/baseline was 5 (range, 0–14) days and 31% of participants were enrolled in the study within 48 hours of diagnosis. Of the participants enrolled, 52% had evidence of being infected within 6 months of the screening/baseline visit and 32% were believed to have been infected for >6 months prior to entering the study. Most participants had WHO clinical stage 1 (asymptomatic) HIV infection (85%) and US Centers for Disease Control and Prevention stage A disease classification (92%).

**Table 1.** Baseline Participant Demographic and Clinical Characteristics

Characteristic	D/C/F/TAF (N = 109)
Demographic characteristics	
Age, y, median (range)	28 (19–66)
Male sex	95 (87)
Race	
White	65 (60)
Black/African American	35 (32)
Other	9 (8)
Body weight, kg, median (range)	78.8 (46–155)
Clinical characteristics	
HIV-1 RNA, No.	108^a^
Median (range), copies/mL	38 700 (19^b^–144 000 000)
≥100 000 copies/mL	27 (25)
CD4^+^ cell count, No.	108^a^
Median (range), cells/µL	369 (7–1082)
<200 cells/µL	23 (21)
HIV acquisition risk factor^c^	
Heterosexual contact	17 (16)
Intravenous drug use	2 (2)
MSM	82 (75)
Multiple	5 (5)
Other	3 (3)
Time from diagnosis to screening/baseline, d, median (range)	5 (0–14)
Enrolled within 48 h of diagnosis	34 (31)
Duration of infection, No.^c^	108^b^
Acute infection^d^	13 (12)
Early infection^e^	43 (40)
Chronic infection^f^	34 (32)
Unknown	18 (17)
WHO clinical stage of HIV infection	
Stage 1 (asymptomatic)	93 (85)
Stage 2 (mild symptoms)	11 (10)
Stage 3 (advanced symptoms)	5 (5)
CDC disease classification^c^	
Stage A	100 (92)
Stage B	6 (6)
Stage C	3 (3)
General characteristics^g^	
Active nicotine use	45 (41)
Active alcohol consumption	90 (83)
Insurance coverage	67 (61)
Employment status^c^	
Employed^h^	87 (80)
Unemployed	16 (15)
Other^i^	6 (6)
Social support^c^	
Case manager^j^	4 (4)
Family/friends/multiple	100 (92)
Missing	5 (5)
Current housing situation^c^	
Own	17 (16)
Rent	51 (47)
Live with friends/family or other	41 (38)

Data are presented as no. (%) unless otherwise indicated.

Abbreviations: CDC, Centers for Disease Control and Prevention; D/C/F/TAF, darunavir/cobicistat/emtricitabine/tenofovir alafenamide; HIV, human immunodeficiency virus; HIV-1, human immunodeficiency virus type 1; MSM, men who have sex with men; WHO, World Health Organization.

^a^One participant had missing values due to a shipping error of the screening/baseline samples.

^b^One participant was HIV-1 negative (false-positive fourth-generation test).

^c^Percentages may not total 100% due to rounding.

^d^Acute infection was defined as HIV-1 antibody negative and HIV-1 RNA positive/ p24 positive.

^e^Early infection was defined as HIV-1 antibody positive and suspected infection ≤6 mo prior to screening/baseline.

^f^Chronic infection was defined as HIV-1 antibody positive and suspected infection >6 mo prior to screening/baseline.

^g^The most common (≥15% of participants) medical history events were seasonal allergy (24%), hypertension (17%), anxiety (17%), and syphilis (16%).

^h^Employed includes employed full time for wages, employed part time for wages, and self-employed.

^i^Other includes retired, short- or long-term disability, student, and other.

^j^Case manager who helps with medication administration.

Among the participants with available genotype data from screening/baseline, no darunavir or tenofovir RAMs were observed, and all participants exhibited full genotypic susceptibility to darunavir and tenofovir (Table 2). Two participants had emtricitabine RAMs (M184M/I and M184M/V) and 5 had a primary PI RAM, but none were darunavir RAMs. Five participants were found to have a transmitted INI mutation at position T97.

**Table 2.** Human Immunodeficiency Virus Type 1 Genotype at Screening/Baseline

Genotype Parameter	D/C/F/TAF (n = 102)^a^
Genotypic susceptibility	
Darunavir	102 (100)
Emtricitabine	100 (98)
Tenofovir	102 (100)
All PIs	97 (95)
All NRTIs	98 (96)
All NNRTIs	80 (78)
All INIs	97 (95)
≥1 RAM	
Primary PI	5 (5)
Secondary PI	100 (98)
Darunavir	0
Emtricitabine	2 (2)
M184M/I	1 (<1)
M184M/V	1 (<1)
Tenofovir	0
NNRTI^b^	28 (28)
K103N	11 (11)
Primary INI	0
Secondary INI	5 (5)
T97T/A	3 (3)
T97A	2 (2)

Data are presented as no. (%).

Abbreviations: D/C/F/TAF, darunavir/cobicistat/emtricitabine/tenofovir alafenamide; INI, integrase inhibitor; NNRTI, nonnucleoside reverse transcriptase inhibitor; NRTI, nucleoside reverse transcriptase inhibitor; PI, protease inhibitor; RAM, resistance-associated mutation.

^a^Genotypes were not available for 7 participants due to being unable to amplify (ie, low viral load, reduced viral fitness, compromised sample collection/handling, primer incompatibility).

^b^Individual NNRTI RAMs are only shown for those occurring in ≥10% of participants.

### Participant Disposition

Overall, 97 (89%) participants completed the study and 12 (11%) discontinued by week 48 (see Table 3 for reasons). No participants discontinued due to protocol-defined resistance stopping rules. Among participants with early discontinuation who completed a retention assessment, 6 of 7 (86%) remained engaged in care. Three of 5 participants with confirmed transaminase elevations ≥2.5 times the upper limit of normal at the screening/baseline visit, prior to starting D/C/F/TAF, discontinued due to safety stopping criteria. The remaining 2 participants continued based on the investigator’s clinical assessment and agreement of the sponsor. Aminotransferase levels appeared to normalize while either receiving up to 16 days of treatment with D/C/F/TAF (for those who discontinued the study) or continuing D/C/F/TAF through the study period (see Supplementary Table 2 for clinical summaries).

**Table 3.** Participant Disposition Through Week 48

Disposition	D/C/F/TAF (N = 109)
Completed	97 (89)
Discontinued^a^	12 (11)
Baseline resistance	0 (0)
Safety stopping rules	3 (3)
Adverse event	1 (<1)
Lost to follow-up	4 (4)
Protocol violation	1 (<1)
Withdrawal of consent	1 (<1)
Other^b^	2 (2)

Data are presented as no. (%).

Abbreviations: D/C/F/TAF, darunavir/cobicistat/emtricitabine/tenofovir alafenamide.

^a^Of the 11 participants who prematurely discontinued treatment, retention in care assessment was completed for 7 (64%) participants; among these participants, 6 (86%) had a documented clinical visit within 90 days of discontinuing D/C/F/TAF. One participant withdrew consent and no follow-up effort was made, while 1 participant was lost to follow-up (the site attempted a follow-up that yielded no information [failed attempt]).

^b^Other reasons were participant incarceration and switch to another antiretroviral due to D/C/F/TAF food requirements.

### Efficacy

**Figure 1.**
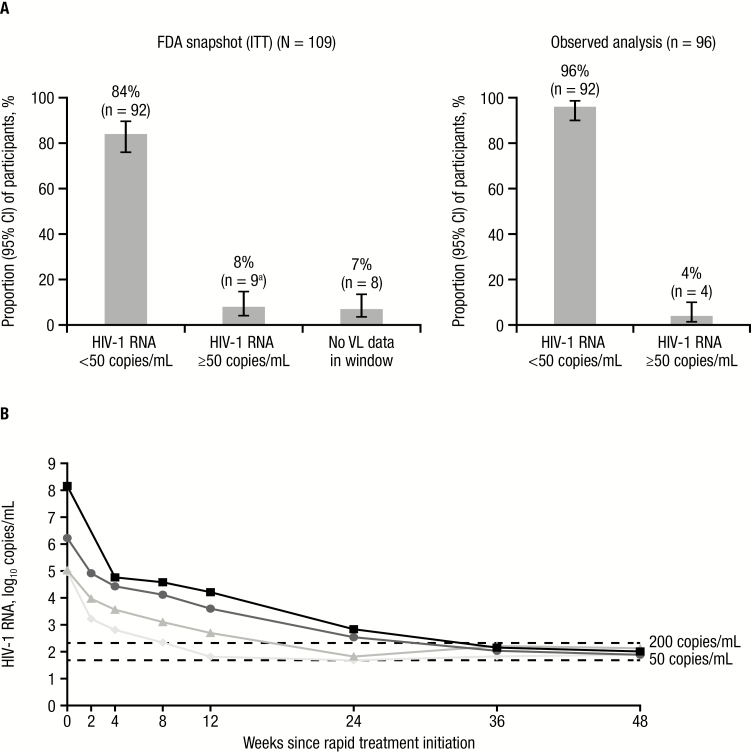
Darunavir/cobicistat/emtricitabine/tenofovir alafenamide virologic efficacy in a rapid-initiation model of care. *A*, Virologic response at week 48. ^a^Three participants discontinued early due to protocol-defined safety stopping rules. *B*, Log_10_ human immunodeficiency virus type 1 (HIV-1) RNA over time for individual participants with HIV-1 RNA ≥50 copies/mL at week 48 (observed analysis; n = 4). HIV-1 RNA level was not available for 1 participant at the week 2 visit. The participant with HIV-1 RNA 144 000 000 copies/mL at screening/baseline was a 30-year-old black/African American man with a CD4^+^ cell count of 242 cells/µL, Centers for Disease Control and Prevention (CDC) classification stage A, World Health Organization (WHO) clinical stage 1 (asymptomatic), and acute infection. The participant with HIV-1 RNA 1 680 000 copies/mL at screening/baseline was a 54-year-old white man with a CD4^+^ cell count of 8 cells/µL, CDC classification stage A, WHO clinical stage 1 (asymptomatic), and chronic infection. The participant with HIV-1 RNA 105 000 copies/mL at screening/baseline was a 28-year-old white man with a CD4^+^ cell count of 468 cells/µL, CDC classification stage A, WHO clinical stage 1 (asymptomatic), and early infection. The participant with HIV-1 RNA 92 900 copies/mL at screening/baseline was a 63-year-old black/African American woman with a CD4^+^ cell count of 127 cells/µL, CDC classification stage B, WHO clinical stage 2 (mild symptoms), and chronic infection. Abbreviations: CI, confidence interval; FDA, Food and Drug Administration; HIV-1, human immunodeficiency virus type 1; ITT, intent to treat; VL, viral load.

At week 48, 92 of 109 (84%) participants achieved HIV-1 RNA <50 copies/mL (FDA snapshot [ITT]); 9 (8%) participants had HIV-1 RNA ≥50 copies/mL (including 5 who discontinued early due to other reasons; the remaining 4 participants had HIV-1 RNA <200 copies/mL), and 8 (7%) participants did not have viral load (VL) data in the week 48 window (Figure 1A). No participants discontinued due to lack of efficacy or developed PDVF. Both participants with M184V/I mutations at screening/baseline achieved HIV-1 RNA <50 copies/mL by week 4; thereafter, 1 participant discontinued early as a result of switching ART regimen (due to D/C/F/TAF food requirements) and the other participant had a VL that remained undetectable through week 48.

**Figure 2.**
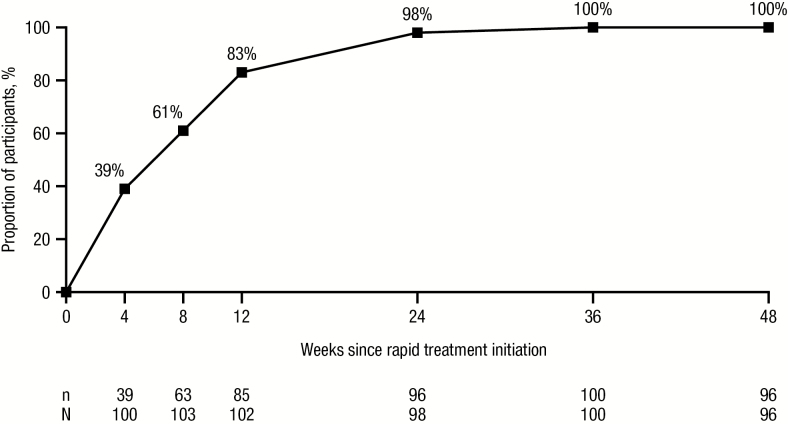
Virologic response over time since darunavir/cobicistat/emtricitabine/tenofovir alafenamide rapid initiation (human immunodeficiency virus type 1 RNA <200 copies/mL; observed analysis).

According to the observed analysis, 92 of 96 (96%) participants achieved HIV-1 RNA <50 copies/mL at week 48 (Figure 1A); the remaining 4 participants had HIV-1 RNA <200 copies/mL (Figure 1B). The threshold of HIV-1 RNA <200 copies/mL (observed analysis) was reached by 85 of 102 (83%) participants at week 12 and 96 of 98 (98%) participants by week 24 (Figure 2). Efficacy was consistent across a variety of baseline demographic and clinical characteristics (Supplementary Table 3). Overall, the mean CD4^+^ cell count was 413 (standard error [SE], 24) cells/µL at screening/baseline and 628 (SE, 30) cells/µL at week 48.

### Safety

Overall, most AEs were grade 1 or 2 in severity and incidences of grade 3 or 4 AEs were low (Table 4). There were no serious or grade 4 AEs that were considered to be study drug related, and there were no deaths. Two grade 3 AEs were considered to be study drug related: allergic dermatitis (accompanied by pyrexia [grade 2] and lip swelling [grade 2]), which resolved after discontinuation of study treatment; and nausea, which resolved with no changes in study drug dosing. The most common (≥2% of participants; any grade) ADRs were diarrhea (12%), nausea (12%), rash (5%), vomiting (4%), and fatigue (3%), and most ADRs were grade 1. There were no reports of immune reconstitution inflammatory events and no discontinuations due to central nervous system, gastrointestinal, metabolic, renal, or bone AEs.

**Table 4.** Summary of Adverse Events and Adverse Drug Reactions Through Week 48

	D/C/F/TAF (N = 109)	
Adverse Event	Overall	At Least Possibly Related
Any	92 (84)	36 (33)
Serious	10 (9)	0
Grade 1	30 (28)	27 (25)
Grade 2	48 (44)	7 (6)
Grade 3	13 (12)	2 (2)
Grade 4^a^	1 (<1)	0
Most common ADRs (≥2% of participants)	Any Grade	≥Grade 2
Diarrhea	13 (12)	2 (2)
Nausea	13 (12)	2 (2)
Rash^b,c^	5 (5)	4 (4)
Vomiting	4 (4)	0
Fatigue	3 (3)	0

Data are presented as no. (%).

Abbreviations: ADR, adverse drug reaction; D/C/F/TAF, darunavir/cobicistat/emtricitabine/tenofovir alafenamide.

^a^Abdominal injury (grade 4, not related) secondary to motor vehicle accident (grade 3, not related).

^b^Pooled preferred terms of allergic dermatitis, dermatitis, rash, macular rash, maculopapular rash, papular rash, and pruritic rash.

^c^All rash adverse events were grade 1 or 2, except for 1 that was grade 3.

There was a median increase in body weight from baseline through week 48 of 2.9 (95% bootstrap confidence interval, 1.5–4.1) kg (mean increase, 4.3 kg). Few grade 3 and 4 laboratory abnormalities occurred in ≥2% of participants; those that did were increased bilirubin (3 [3%] participants), increased alanine aminotransferase (3 [3%] participants), and increased aspartate aminotransferase (5 [5%] participants); all of these were asymptomatic and none warranted treatment discontinuation according to the protocol as none were deemed to be related to study drug.

### Patient-reported Outcomes

**Figure 3.**
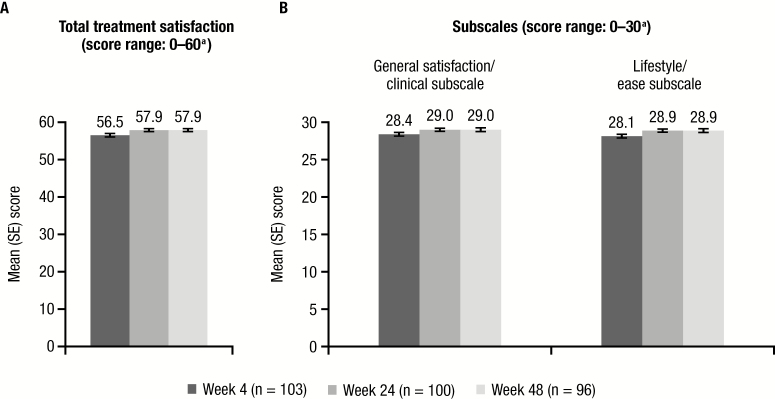
Human Immunodeficiency Virus Treatment Satisfaction Questionnaire status version (HIVTSQs) scores at weeks 4, 24, and 48 after rapid initiation of darunavir/cobicistat/emtricitabine/tenofovir alafenamide. *A*, Total HIVTSQs scores. *B*, HIVTSQs subscale scores. ^a^Higher scores indicate greater satisfaction. Abbreviation: SE, standard error.

Responses to the HIVTSQs indicated high levels of total treatment satisfaction for participants rapidly initiating and continuing D/C/F/TAF, with scores that approached the maximum of 60 at weeks 24 and 48 (Figure 3A); correspondingly, scores on the general satisfaction/clinical and lifestyle/ease subscales were also high at both timepoints (Figure 3B). At week 48, a majority of participants reported they were satisfied (score of 5 or 6) with their treatment (97%) and would recommend (score of 5 or 6) their present treatment to someone else with HIV (98%). Participant responses to all 10 HIVTSQs questions are summarized in Supplementary Figure 2.

## DISCUSSION

In this prospective study of an STR in a rapid-initiation model of care, a high proportion (89%) of participants continued D/C/F/TAF treatment through week 48, and rates of virologic response (HIV-1 RNA <50 copies/mL) were high (84%–96%). No participants discontinued due to lack of efficacy or met PDVF criteria. The low discontinuation rates due to tolerability issues and the high treatment satisfaction scores observed in this study yielded a large proportion of the population who remained on treatment through week 48; among these participants, >90% (of those with data available) achieved virologic response. These retention and suppression rates, as well as the observed 86% retention in care among participants with early discontinuation, are aligned closely with WHO 90-90-90 goals [16].

Transmitted resistance is an important consideration for patients newly infected with HIV-1 because it could influence choice of ART regimen; in rapid-initiation settings, this information is not available prior to starting therapy. In DIAMOND, while 5 participants had ≥1 primary PI RAM, none had darunavir RAMs. Additionally, 2 participants had an M184I/V mutation (associated with emtricitabine resistance), both of whom achieved virologic suppression after rapid initiation with D/C/F/TAF. While rarely observed to date, transmitted resistance to the integrase class was observed in 5 participants with INI mutations at T97. Notably, a recent study suggested that T97A may be considered a primary INI RAM, affecting sensitivity to raltegravir and elvitegravir and, when in combination with other INI mutations, dolutegravir and bictegravir [17, 18].

Various US centers have implemented rapid-initiation programs (eg, Rapid ART Program for Individuals with HIV Diagnosis [RAPID], CrescentCare Start Initiative, Rapid Entry and ART in Clinic for HIV [REACH], JumpStart), but studies are limited [8–10, 19]. In a retrospective cohort analysis, 95.8% of patients had achieved HIV-1 RNA <200 copies/mL at least once after 1 year of treatment [8]. In another study, viral suppression (HIV-1 RNA <200 copies/mL) was maintained in 70 of 71 patients by the end of the study period [9]. Both of these rapid-initiation studies enrolled a diverse patient population with numerous comorbidities and were conducted in a single, large urban center. In contrast, a diverse set of study sites was selected for DIAMOND in an effort to enroll a population representative of US patients with HIV-1, given the disproportionate impact of HIV-1 on the MSM and black/African American communities and the southern US [20, 21].

D/C/F/TAF has shown efficacy and safety in treatment-naïve patients [11, 22, 23] and has characteristics of an ideal regimen for rapid initiation. The demonstrated high genetic barrier to resistance [13, 24] is important given that laboratory test results are not available when treatment is rapidly initiated, and the STR formulation is preferable given evidence of higher adherence rates compared with multitablet regimens [25, 26]. Furthermore, D/C/F/TAF has shown a favorable tolerability profile [11, 12]; these findings were confirmed in DIAMOND. Most AEs were grade 1 or 2, and there were no reports of immune reconstitution inflammatory syndrome AEs. This is noteworthy because 23 (21%) participants had CD4^+^ cell count <200 cells/µL and 27 (25%) had HIV-1 RNA ≥100 000 copies/mL at screening/baseline, and HIV-1 RNA levels decreased to <200 copies/mL soon after beginning treatment.

Recently, body weight–related concerns regarding the use of INIs in combination with or without TAF have been raised. Randomized controlled trials over 48 weeks have demonstrated a mean weight increase of 3 kg [27] and a median increase of 5 kg [28] for patients receiving dolutegravir with a tenofovir disoproxil fumarate–based backbone, and a mean increase of 6 kg for those receiving dolutegravir in combination with a TAF-based backbone [27]. While some change in body weight may be attributed to a “return to health” effect, evidence suggests these increases may be progressive over time. In patients receiving either bictegravir or dolutegravir with a TAF-based backbone, body weight increased over 48 weeks of treatment and continued to increase thereafter, with median changes ranging from 3.5 (bictegravir) to 3.9 kg (dolutegravir) over 96 weeks [29]. In contrast, in a larger study of D/C/F/TAF, the mean and median weight change in treatment-naïve patients over 96 weeks was 2.3 kg and 2.0 kg, respectively (unpublished data). Overall, the effects of different antiretroviral agents on weight may be an important factor to consider when selecting a regimen given the possible impacts on long-term health.

DIAMOND evaluated PROs of ART rapid initiation using the HIVTSQs, which has previously been used for treatment-naïve patients (although not in a rapid-initiation scenario). In 1 study, treatment-naïve patients reported median total HIVTSQs scores of 57–58 (out of 60) 48 weeks after initiating treatment with a multitablet darunavir- or dolutegravir-based regimen [30]. Another study reported median total HIVTSQs scores of 53–55 (out of 60) 96 weeks after initiating treatment with either once-daily darunavir/twice-daily raltegravir or once-daily darunavir with tenofovir/emtricitabine [31]. In DIAMOND, the mean HIVTSQs total score was 58 at both weeks 24 and 48. These findings suggest that patients newly diagnosed with HIV-1 infection who rapidly initiate D/C/F/TAF can achieve and maintain high treatment satisfaction scores through 48 weeks. Moreover, results consistent with the overall population were observed for the subgroup of black/African American participants [32].

A limitation of this study is that participants were motivated to start ART and had access to clinical trial–related services (eg, transportation, no-cost ART). Additional work is needed to better understand and mitigate systemic barriers to treatment faced by patients in clinical experience. Notably, while overall retention in DIAMOND was high, participants who started treatment within 24–48 hours of enrollment were more likely than those who started later to be retained in care [33]. Additional study limitations that limit generalizability include the nonrandomized, noncomparative design; variations in treatment implementation by study site; that the number of patients unwilling to participate could not be quantified; and the exclusion of patients with certain AIDS-related conditions and the small proportion of women who enrolled.

In DIAMOND, 12 weeks after rapid initiation of D/C/F/TAF, a majority of participants with available data (>80%) achieved HIV-1 RNA <200 copies/mL, a threshold recognized by multiple organizations, such as the Prevention Access Campaign’s “Undetectable = Untransmittable” and the US DHHS, as the threshold at which patients are unable to transmit HIV to uninfected sexual partners [34, 35]. Moreover, as an STR, D/C/F/TAF may improve treatment adherence, a critical component of maintaining viral suppression and reducing transmission [25, 26, 36]. Taken together, the efficacy, safety, and PRO results from DIAMOND support D/C/F/TAF as a recommended regimen for rapid initiation in most treatment guidelines [2, 4].

## Supplementary Data

Supplementary materials are available at *Clinical Infectious Diseases* online. Consisting of data provided by the authors to benefit the reader, the posted materials are not copyedited and are the sole responsibility of the authors, so questions or comments should be addressed to the corresponding author.

ciz1213_suppl_Supplementary_Material

## Notes

***Author contributions.*** G. D. H., G. C., M. R., J. G., and R. B. contributed to the conduct of the study as investigators and to the interpretation of the data. D. L. contributed to statistical analysis and interpretation of the data. R. B. S., R. E. N., C. B., and K. D. contributed to the design of the study and interpretation of the data. All authors contributed to drafting the manuscript and approved the final version.

***Acknowledgments.*** Medical writing support was provided by Dan Jackson, PhD, and Courtney St. Amour, PhD, of MedErgy, and was funded by Janssen Scientific Affairs, LLC.

***Financial support.*** This work was supported by Janssen Scientific Affairs, LLC.

***Potential conflicts of interest.*** G. D. H. has received research or grant support from Gilead, ViiV, Janssen, Proteus, and the US National Institutes of Health. G. C. has received grants and speaker’s fees from Gilead, Janssen, ViiV, and Merck. M. R. has served as a speaker for Gilead, AbbVie, Janssen, ViiV, and Cubist and has served on advisory boards for Bristol-Myers Squibb (BMS), Merck, and Gilead. J. G. has been a consultant or speaker in conferences supported by AbbVie, BMS, GlaxoSmithKline (GSK), ViiV, Janssen, Merck, and Gilead; is affiliated with an institution that received research grants from AbbVie, BMS, GSK, ViiV, Boehringer Ingelheim, Pfizer, Janssen, Merck, and Gilead; and has served as an investigator for Abbott, Avexa, Boehringer Ingelheim, Gilead, GSK, Merck, Pfizer, Roche, Parexel, Hiesped, and Janssen. D. L., R. B. S., R. E. N., C. B., and K. D. are full-time employees of Janssen and may be stockholders in Johnson & Johnson. R. B. reports no potential conflicts of interest. All authors have submitted the ICMJE Form for Disclosure of Potential Conflicts of Interest. Conflicts that the editors consider relevant to the content of the manuscript have been disclosed.
