# Supplementary material for: Darunavir/Cobicistat/Emtricitabine/Tenofovir Alafenamide in a Rapid-Initiation Model of Care for Human Immunodeficiency Virus Type 1 Infection: Primary Analysis of the DIAMOND Study
Source: Clin Infect Dis. 2019 Dec 27;71(12):3110–7. doi: 10.1093/cid/ciz1213 (PMC7819515; doi:10.1093/cid/ciz1213)
Supplement: ciz1213_suppl_Supplementary_Material [file ciz1213_suppl_supplementary_material.docx]

**Supplemental Materials**

**Methods**

***Study Population***

The key inclusion criterion of antiretroviral treatment–naïve excluded use of emtricitabine/tenofovir disoproxil fumarate for pre-exposure prophylaxis.

Key exclusion criteria were: presence of select opportunistic infections (active cryptococcal infection, active toxoplasmic encephalitis, or *Mycobacterium tuberculosis* infection), or another acquired immunodeficiency syndrome (AIDS)–defining condition that in the investigator’s judgment would increase the risk of morbidity/mortality; known history of clinically relevant hepatic disease or hepatitis that in the investigator’s judgment is not compatible with darunavir/cobicistat/emtricitabine/tenofovir alafenamide (D/C/F/TAF); cirrhosis; and chronic (≥3 months) renal insufficiency, defined as glomerular filtration rate (eGFR; according to the Modification of Diet in Renal Disease [MDRD] formula) <50 mL/min.

Safety stopping criteria included: eGFR ([MDRD] formula) <50 mL/min; aspartate aminotransferase or alanine aminotransferase ≥2.5 times the upper limit of normal (ULN); serum lipase ≥1.5 times the ULN; positive pregnancy test for women of childbearing potential; laboratory results that the investigator believes should result in discontinuation of study medication; and active hepatitis C infection that, in the opinion of the investigator, required immediate treatment or was expected to require treatment during the study with agents not compatible with D/C/F/TAF. Retesting of abnormal screening/baseline safety laboratory values was allowed once; retesting took place at an unscheduled visit.

In determining the duration of infection, the following definitions were used: acute infection was defined as human immunodeficiency virus (HIV)–1 antibody negative and HIV-1 RNA positive/p24 positive, early infection was defined as HIV-1 antibody positive and suspected infection ≤6 months prior to screening/baseline, and chronic infection was defined as HIV-1 antibody positive and suspected infection >6 months prior to screening/baseline.

***Analyses***

Absolute CD4+ cell count at screening/baseline and Week 48 were described as additional measures to assess efficacy.

Retention in care was only assessed for participants who withdrew from the study prematurely (ie, prior to Week 48 visit window [Weeks 42-54]) for any reason, including loss to follow-up. Retention in care was defined as a documented clinic visit with a healthcare provider within 90 days of discontinuation before Week 48 (unless consent was withdrawn).

Protocol-defined virologic failure was defined as 1 of the following: virologic nonresponse (HIV-1 RNA <1 log_10_ reduction from baseline and ≥400 copies/mL at the Week 12 visit [confirmed within 2-4 weeks]); virologic rebound (at any visit, after achieving confirmed consecutive HIV-1 RNA <50 copies/mL, a rebound to ≥50 copies/mL [confirmed within 2-4 weeks], or, at any visit, a >1 log_10_ increase in HIV-1 RNA from nadir [confirmed within 2-4 weeks]).

***Statistical Analyses***

Descriptive statistics for absolute HIVTSQs scores were calculated for overall treatment satisfaction, both subscales (general satisfaction/clinical subscale and lifestyle/ease subscale), and each question; overall and subscale results are reported for each timepoint and question level results are reported for Week 48. In case of a missing item score for the HIV Treatment Satisfaction Questionnaire status version, the participant’s overall score was calculated as follows: sum the existing score items, divide the sum by the number of existing scores, and then multiply by 10.

**Supplemental Table S1. Geographic Distribution of Participants by State^a^**

| **State, n (%)** | **D/C/F/TAF**  **N = 109** |
| --- | --- |
| Arizona | 8 (7) |
| California | 17 (16) |
| Washington DC | 7 (6) |
| Florida | 22 (20) |
| Georgia | 1 (1) |
| Illinois | 5 (5) |
| Maryland | 1 (1) |
| New Jersey | 2 (2) |
| New Mexico | 7 (6) |
| Oklahoma | 1 (1) |
| Texas | 35 (32) |
| Virginia | 3 (3) |

^a^Study sites were located in the following cities: Phoenix (Arizona); Bakersfield and Los Angeles (California); Washington DC; Fort Pierce, Miami, and Orlando (Florida); Savannah (Georgia); Chicago (Illinois); Newark (New Jersey); Albuquerque and Santa Fe (New Mexico); and Dallas, Fort Worth, and Houston (Texas).

**Supplemental Table S2. Clinical Summary of Participants Who Met Safety Stopping Criteria**

|  | **Hepatitis serology, –/+** | | | |  | **Screening/baseline** | | |  |
| --- | --- | --- | --- | --- | --- | --- | --- | --- | --- |
| **Participant** | **HCV Ab** | **HBs Ab** | **HBc Ab** | **HBs Ag** | **Relevant medical history** | **Aminotransferase laboratory values, U/L** | **CD4+ cell count, cells/µL** | **HIV-1 RNA, copies/mL** | **Post-baseline aminotransferase laboratory values, U/L** |
| 1^a^ | – | – | – | – | Alcoholic hepatitis without ascites | Baseline: AST, 114  Day 10 retest:  AST, 103 | 150 | 34,200 | Day 15 ESTD:  AST, 69 |
| 2^b^ | – | – | – | – | Gastritis, oral thrush | Baseline: AST, 299; ALT, 188  Day 10 retest:  AST, 140; ALT, 128 | 17 | 445,000 | Day 16 ESTD:  AST, 112; ALT, 116 |
| 3 | +^c^ | + | + | – | None | Baseline: AST, 171  Retest:  AST, 183 | 151 | 17,000 | Week 4:  AST, 69  Week 12:  AST, 93  Week 24:  AST, 59  Week 36:  AST, 50  Week 48:  AST, 65 |
| 4^d^ | – | + | + | – | None | Baseline: AST, 113; ALT, 183  Day 3 retest: AST, 84; ALT, 151 | 226 | 311,000 | Day 14 ESTD:  AST, 53 ALT, 71 |
| 5 | – | + | + | – | Recent secondary syphilis infection | Baseline: AST, 118; ALT, 146  Retest: AST, 70; ALT, 123 | 242 | 144,000,000 | Week 2:  AST, 15; ALT, 28  Week 4:  AST, 14; ALT, 15  Week 8:  AST, 17; ALT, 23  Week 12:  AST, 18; ALT, 24  Week 24:  AST, 14; ALT, 16  Week 36:  AST, 18; ALT, 19  Week 48:  AST, 17; ALT, 21 |

HCV, hepatitis C virus; Ab, antibody; HBs, hepatitis B surface; HBc, hepatitis B core; Ag, antigen; HIV-1, human immunodeficiency virus–1; AST, aspartate aminotransferase; ESTD, early study treatment discontinuation; ALT, alanine aminotransferase.

^a^Participant discontinued treatment on Day 15.

^b^Participant discontinued treatment on Day 15.

^c^HCV RNA tested negative.

^d^Participant discontinued treatment of Day 13.

**Supplemental Table S3. Virologic Response (HIV-1 RNA <50 Copies/mL) at Week 48 by Subgroups**

| **Population** | **FDA snapshot-ITT**  **(N = 109)** | **Observed analysis**  **(n = 96)** |
| --- | --- | --- |
|  | HIV-1 RNA  <50 copies/mL, n/N (%) | |
| Overall | 92/109 (84) | 92/96 (96) |
| Time from diagnosis to enrollment, days^a^ |  |  |
| 0-1 | 21/23 (91) | 21/22 (96) |
| 1-2 | 10/11 (91) | 10/10 (100) |
| 2-3 | 3/4 (75) | 3/3 (100) |
| 3-7 | 32/38 (84) | 32/34 (94) |
| 7-14 | 26/33 (79) | 26/27 (96) |
| Baseline HIV-1 RNA, copies/mL^a,b^ |  |  |
| ≥100,000 | 19/27 (70) | 19/22 (86) |
| <100,000 | 72/81 (89) | 72/73 (99) |
| Baseline CD4+ cell count, cells/µL^a,b^ |  |  |
| ≤200 | 17/23 (74) | 17/19 (90) |
| >200 | 74/85 (87) | 74/76 (97) |
| Age, years |  |  |
| 18-25 | 32/38 (84) | 32/32 (100) |
| 26-50 | 50/58 (86) | 50/52 (96) |
| >50 | 10/13 (77) | 10/12 (83) |
| Gender |  |  |
| Women | 9/14 (64)^c^ | 9/10 (90) |
| Men | 83/95 (87) | 83/86 (97) |
| Race/ethnicity |  |  |
| White | 55/65 (85) | 55/58 (95) |
| Black/African American | 29/35 (83) | 29/30 (97) |
| Other | 8/9 (89) | 8/8 (100) |
| Hispanic | 42/48 (88) | 42/44 (95) |
| Non-Hispanic | 50/61 (82) | 50/52 (96) |

HIV-1, human immunodeficiency virus–1; FDA, Food and Drug Administration; ITT, intent-to-treat.

^a^Differences in virologic response rates (FDA snapshot-ITT) across subgroups were largely driven by early study discontinuation [1].

^b^One participant had missing values due to a shipping error of the screening/baseline samples.

^c^Among the 5 women who did not achieve HIV-1 RNA <50 copies/mL at Week 48, 1 had HIV-1 RNA ≥50 copies/mL (77 copies/mL) at Week 48, 1 had HIV-1 RNA ≥50 copies/mL at early discontinuation, and 3 did not have data in the window.

**Supplemental Figure S1. DIAMOND study design.**

**
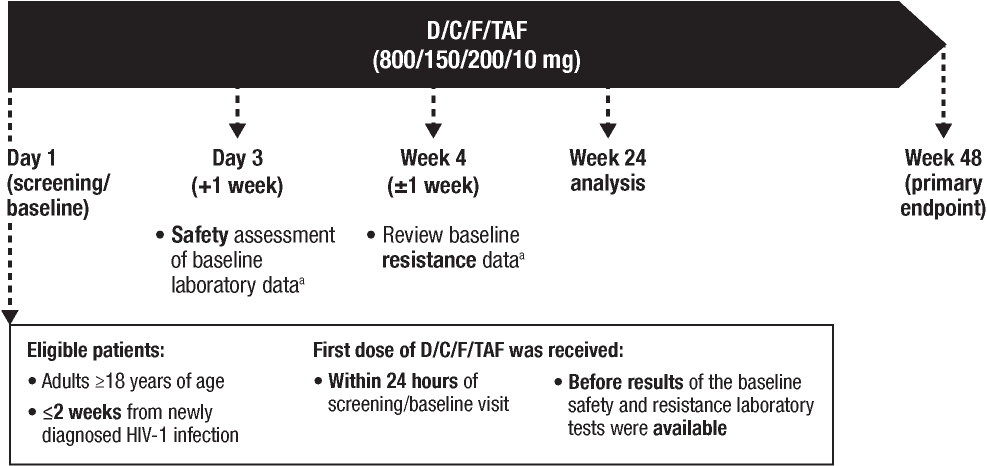
**

D/C/F/TAF, darunavir/cobicistat/emtricitabine/tenofovir alafenamide; HIV-1, human immunodeficiency virus–1.

^a^Evaluations could be performed sooner based on the availability of results.

**Supplemental Figure S2. Participant responses to all HIVTSQs questions at Week 48 after rapid initiation of D/C/F/TAF.^a^**


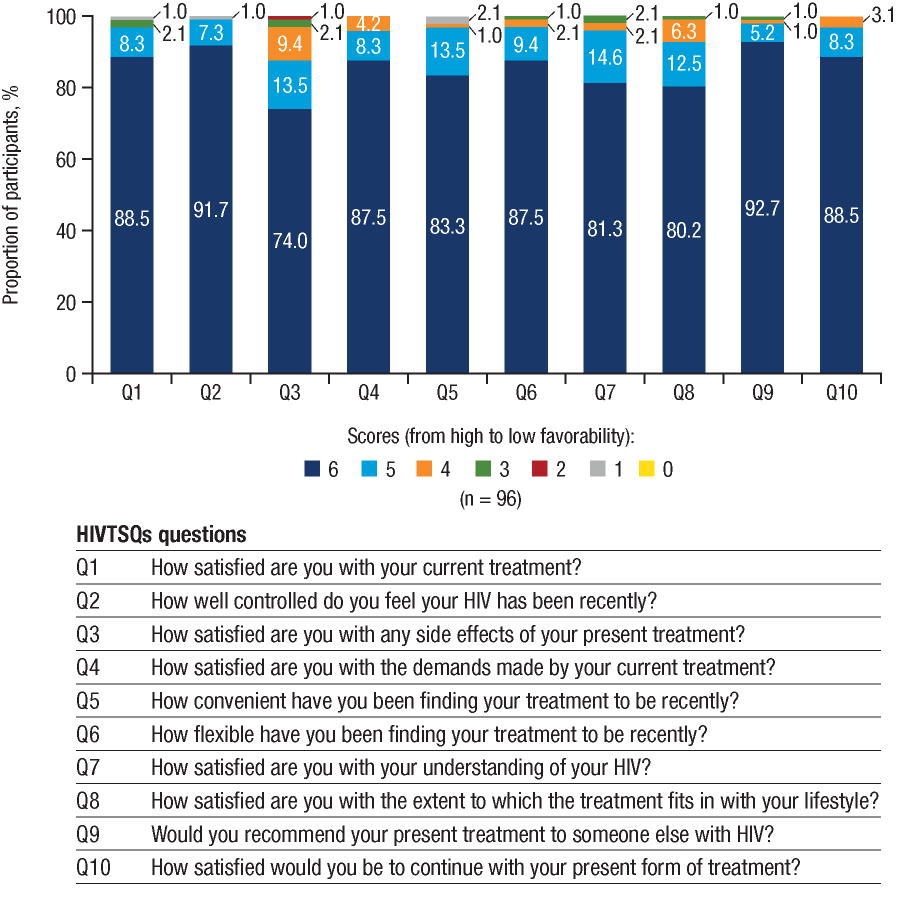


HIVTSQs, HIV Treatment Satisfaction Questionnaire status version; D/C/F/TAF, darunavir/cobicistat/emtricitabine/tenofovir alafenamide; HIV-1, human immunodeficiency virus–1.

^a^Percentages may not total 100.0% due to rounding.

**References**

1. Huhn G, Ramgopal M, Grofoot G, et al. High rates of virologic suppression achieved in HIV-1–infected adults rapidly starting antiretroviral therapy (ART) with the single-tablet regimen (STR) of darunavir/cobicistat/emtricitabine/tenofovir alafenamide (D/C/F/TAF) 800/150/200/10 mg regardless of baseline disease characteristics: Week 48 subgroup analyses from the phase 3 DIAMOND trial. Oral presenation at: IDWeek^TM^ 2019; 2-6 October, 2019; Washington, DC.
